# Influence of Factor V Leiden Mutation and Protein C and Protein S Deficiencies on Preeclampsia Among Sudanese Women

**DOI:** 10.7759/cureus.79969

**Published:** 2025-03-03

**Authors:** Faris Abdon, Maha Elamin, Khalid Bakheit

**Affiliations:** 1 Medical Sciences Department/Medical Biochemistry Unit, Orotta College of Medicine and Health Sciences, Asmara, ERI; 2 Biochemistry Department, Faculty of Medicine and Health Sciences, Al Neelain University, Khartoum, SDN; 3 Clinical Biochemistry Department, King Abdulaziz University Faculty of Medicine, Jeddah, SAU

**Keywords:** factor v leiden, preeclampsia, protein c, protein s, sudan, thrombophilia

## Abstract

Background

Preeclampsia (PE) is a serious pregnancy complication that can endanger the health of both mothers and fetuses. Genetic factors, such as mutations in thrombophilia and deficiencies in natural anticoagulants, could contribute to its development, but their exact roles are not well-understood, especially among Sudanese women.

Objective

To assess the relationship between the Factor V Leiden (FVL) thrombophilic mutation and reduced levels of the natural anticoagulants Protein C (PC) and Protein S (PS) with the occurrence of PE among Sudanese women.

Methods and materials

A case-control study included 300 women, divided equally into three groups: 100 with PE, 100 healthy normotensive pregnant women, and 100 healthy non-pregnant women. Polymerase chain reaction-restriction fragment length polymorphism (PCR-RFLP) was used to detect FVL mutations. PC and PS levels were measured using colorimetric assays. Logistic regression analysis assessed the relationships between these variables and the risk of developing PE.

Results

Our findings showed no significant link between FVL mutations and PE (p=.390). PC levels alone did not emerge as significant independent predictors of PE (OR 1.01, 95% CI 0.99-1.02, p=.419). However, women with low PC and S levels were strongly associated with PE in univariate and multivariate analyses (OR 77.67, 95% CI 8.97-672.5, p<.001). This combination was significantly more common in the PE group than in the control group (p<.001). Additionally, reduced PS levels were significantly associated with an increased risk of PE.

Conclusion

Combined PC/PS deficiencies are strongly associated with PE among Sudanese women, indicating a significant role for these natural anticoagulants in the pathogenesis of the disease. An FVL mutation was not significantly associated with PE in this Sudanese population.

## Introduction

Thrombophilia, or an increased tendency to form blood clots, can be due to inherited or acquired factors. Pregnancy naturally elevates the likelihood of clotting, and when combined with thrombophilia, the chance of thrombosis becomes higher [1]. Mutations that result in protein deficiencies, such as PC or PS (loss-of-function mutations), or mutations that increase the activity of clotting factors, such as FVL (gain-of-function mutations), can cause inherited thrombophilia. Despite some research indicating a correlation between inherited thrombophilia and preeclampsia (PE), this association remains debatable [1].

The most prevalent inherited thrombophilia is FVL, which is caused by a specific mutation (G1691A) in the Factor V gene. This mutation causes a change in amino acids (Arg506Gln) in Factor V (f5), making it resistant to inactivation by activated PC. This resistance results in a hypercoagulable state, particularly during pregnancy [2,3]. The FVL mutation has been linked to an elevated risk of PE and pregnancy loss [4]. Meta-analyses have verified a substantial correlation between the FVL mutation and hypertensive disorders of pregnancy, indicating that FVL may be a genetic risk factor for PE [4]. Nevertheless, the correlation between PE and FVL remains a topic of debate. Some studies indicate PE patients have a higher prevalence of thrombophilia disorders than controls; however, they do not establish a direct correlation between FVL and PE [5]. For example, a study on the Kolar population found no significant association between the FVL mutation and PE [6], and others suggest that due to inconsistent findings, routine thrombophilia screening during pregnancy may not be justified [5]. These findings highlight the importance of considering population-specific genetic backgrounds when studying the genetic factors contributing to PE [3]. Genetic predispositions that may contribute to PE among Sudanese women have been identified in research conducted in Sudan. For example, a study discovered a substantial correlation between PE and the FVL G1691A mutation. FVL was detected in 9.6% of preeclamptic women while it was present in only 0.6% of controls [7]. Another study confirmed a significant association between FVL mutations and severe PE, with a mutation prevalence of 16% in cases compared to 0% in controls [8].

PS and PC are essential components of the body's anticoagulant system, and their deficiencies are recognized to elevate the likelihood of thrombotic events [9]. PC is a vitamin K-dependent enzyme that is crucial for the regulation of blood coagulation. It is activated by the thrombin-thrombomodulin complex and, in conjunction with PS, prevents thrombin formation by deactivating coagulation cofactors [9]. PS, also a vitamin K- dependent glycoprotein, is a cofactor for activated PC and has independent anticoagulant properties [9]. PS and PC levels decrease during pregnancy, and any deficiencies can result in a hypercoagulable state, which may contribute to the development of PE [3].

The prevalence and impact of PC and S deficiencies in PE have been the subject of conflicting research results. For example, Igwe et al. reported that Nigerian women with increased pregnancy loss had reduced levels of PC. In contrast, other studies did not observe significant differences in PS levels between preeclamptic and healthy women [10].

PE and other adverse outcomes are significantly increased by consanguinity, notably marriages between cousins. In Lahore, Pakistan, consanguinity was associated with 50% of PE and 70% of eclampsia cases [11]. This practice, common in the Middle East due to sociocultural reasons, also increases the risk of congenital disorders [12]. However, in Iran, consanguineous marriage was linked to an increased risk of stillbirth, particularly preterm stillbirths [13]. Additionally, PE tends to be more severe in consanguineous couples, with higher blood pressure and proteinuria, suggesting a genetic predisposition [14].

In our recent Sudanese case-control study (Abdon et al.), advanced maternal age, lower education, longer marital duration, and a family history of preeclampsia significantly predicted PE. These observations agree with the broader evidence that PE arises from a constellation of demographic, clinical, and biochemical influences [15]. While many of such factors merit investigation, we have chosen to concentrate on the thrombophilic markers Protein C (PC), Protein S (PS), and Factor V Leiden (FVL). Prior local research, such as Elzein et al., indicates that inherited thrombophilias may play a pivotal role in PE development, prompting our focused examination of these markers within Sudan [8].

This article was previously posted to the medRxiv preprint server on November 2, 2024.

## Materials and methods

From 2019 to 2021, a case-control study was conducted at Omdurman Maternity Hospital in Khartoum State, Sudan, to examine the correlation between preeclampsia (PE) and Factor V Leiden (FVL) mutations in Sudanese women, as well as investigate natural blood coagulation inhibitors, including activated Protein C resistance (APC-R). A total of 300 women were enrolled, divided equally into three groups: 100 women diagnosed with PE, 100 healthy normotensive pregnant women, and 100 healthy non-pregnant women.

PE was defined as new-onset hypertension (blood pressure ≥140/90 mmHg on at least two occasions) after 20 weeks of gestation, accompanied by significant proteinuria (>300 mg/dL or ≥2+ on dipstick) or other systemic findings. Women who had hypertension before 20 weeks’ gestation, multiple pregnancies (e.g., twins), or other systemic health conditions were excluded from the PE group. The control group of healthy pregnant women consisted of normotensive individuals with uncomplicated singleton pregnancies, and their expected delivery dates were closely matched to those of the PE group. An additional control group of non-pregnant women was included to establish baseline (pre-pregnancy) Protein C and Protein S levels, thereby distinguishing inherent thrombophilic profiles from pregnancy-induced changes. These non-pregnant controls were non-smokers, had no history of thromboembolic disease, and did not take anticoagulant medications.

Exclusion criteria applied across all groups included a history of chronic hypertension, diabetes, kidney disease, autoimmune disorders, smoking, thromboembolic events, and anticoagulant drug use.

Data collection and biochemical assays

Demographic, clinical, and biochemical information was gathered from each participant using a validated questionnaire. Venous blood samples were collected to extract genomic DNA and assess PC and S levels. PC was activated with a chromogenic substrate, and the absorbance was measured spectrophotometrically to ascertain PC levels. Similarly, PS concentrations were determined by initiating a reaction with thrombin and a chromogenic substrate, which resulted in a change in color intensity that indicated the concentration of PS. PC and PS were assessed for activity levels, with reference ranges of 65%-150% for PC and 57%-131% for PS [16].

Molecular methods

Genomic DNA was extracted from venous blood samples using the G-DEX™ IIb Genomic DNA Extraction Kit for blood (iNtRON Biotechnology) according to the manufacturer’s instructions, and the quantity and purity of the isolated DNA were assessed with a NanoDrop™ spectrophotometer (Thermo Fisher Scientific Inc., Waltham, MA, US). Missense mutations in the F5 gene, specifically the Factor V Leiden (FVL) R506Q polymorphism (G1691A), were then identified using a polymerase chain reaction-restriction fragment length polymorphism (PCR-RFLP) assay. A 206 bp region encompassing nucleotide position 1691 of the F5 gene was amplified with a hot-start Taq polymerase activation step at 95 °C for 10 minutes, followed by 35 cycles of denaturation at 95 °C for 30 seconds, annealing at 55 °C for 1 minute, and extension at 70 °C for 3 minutes, concluding with a final extension at 70 °C for 3 minutes. The forward primer sequence was 5′-TGCCCCATTATTTAGCCAGGAG-3′, and the reverse primer sequence was 5′-ACCCACAGAAAATGATGCCCAG-3′.

After amplification, the 206 bp PCR product was digested with the MnII restriction enzyme (Thermo Scientific, EU) at 37 °C for 3 hours in a total reaction volume of 30 μl, per the manufacturer’s protocol. The digested products were then separated by electrophoresis on 1.5% agarose gels (iNtRON Scientific) and visualized under UV light. The resulting fragment patterns indicated the genotype: two fragments (47 bp and 159 bp) for mutant homozygous (A/A allele), three fragments (136 bp, 47 bp, and 23 bp) for wild-type homozygous (G/G allele), and four fragments (102 bp, 47 bp, 34 bp, and 23 bp) for heterozygous (A/G allele). Each gel was evaluated to confirm whether the individual carried the wild-type genotype, the mutant allele, or a heterozygous combination.

Ethics methods

This study was approved by the Ethics Committee of Al-Neelain University (IRB Serial No: NU-IRB-18-8-8-41) on (30/08/2018). All participants provided written informed consent using standardized consent forms. The research followed the Declaration of Helsinki and adhered to all relevant institutional and national ethical guidelines.

Data analysis

Data were analyzed using IBM SPSS Statistics version 29.0 (IBM Corp., Armonk, NY, US) to identify PE predictors among Sudanese women. The sociodemographic, clinical, and biochemical characteristics of the case and control groups were compared using descriptive statistics. Categorical variables were analyzed using chi-square tests while continuous variables were analyzed using t-tests or Mann-Whitney U tests, as appropriate. Univariate logistic regression analysis was implemented to identify potential predictors of PE, followed by multivariate logistic regression analysis to account for potentially confounding variables. A backward incremental method was used to identify significant predictors. The Hosmer-Lemeshow test and the area under the receiver operating characteristic (ROC) curve (AUC) were used to assess the model’s goodness of fit. Variance inflation factors (VIFs) were examined to check for multicollinearity.

## Results

The median age of women with PE was 36 years, significantly higher than that of the healthy pregnant group (29 years) and the non-pregnant group (31 years), p < 0.001 (Table 1). The non-pregnant group (72%) had a higher prevalence of secondary education than the PE group (58%) and the healthy pregnant group (51%). Employment rates were 23% lower in the PE group as compared to the non-pregnant group. Consanguinity was more common among pregnant women (67% in PE, 65% in healthy pregnant) than in non-pregnant controls (20%). Oral contraceptive use was significantly higher in the PE group (13%) than in the non-pregnant group (1%), p < 0.001.

**Table 1:** Comparison of sociodemographic, clinical, and laboratory parameters among PE cases, healthy pregnant controls, and non-pregnant controls Note: All p-values in this table were determined via chi-square for categorical variables and Mann-Whitney U or t-tests for continuous variables. p < 0.05 was considered significant, and p < 0.001 was considered highly significant. PE: preeclampsia; IQR: interquartile range; PC: Protein C; PS: Protein S

	Cases (N=100)	Controls (N=200)		
Characteristics	(1) Preeclampsia Group	(2) Healthy Pregnant Group	(3) Non-Pregnant Group	p-value
Age in years median (IQR)	36 (31.25-39)	29 (27-32)	31 (27-34)	1vs.2: <.001
				2vs.3: .368
				1vs.3: <.001
Educational Level (Up to Secondary %)	58%	51%	72%	1vs.2: .320
				2vs.3: .002
				1vs.3: .038
Employment (Employed %)	23%	30%	38%	1vs.2: .262
				2vs.3: .232
				1vs.3: .021
Consanguinity	67%	65%	20%	1vs.2: .765
				2vs.3: <.001
				1vs.3: <.001
Contraceptive Pills	13%	9%	1%	1vs.2: .366
				2vs.3: .009
				1vs.3: <.001
Presenting Blood Pressure				
Systolic BP median-IQR	150 (140 - 160)	120 (113 - 120)	120 (115-120)	1vs.2: <.001
				2vs.3: .392
				1vs.3: <.001
Diastolic BP median-IQR	100 (90 - 120)	80 (70 - 80)	80 (80-80)	1vs.2: <.001
				2vs.3: .080
				1vs.3: <.001
Platelets median-IQR	244 (233.3-252.8)	277.5 (184 - 312)	290 (197.5-357.5)	1vs.2: .006
				2vs.3: .409
				1vs.3: .011
Levels of PC and PS				
PC median-IQR	107.8 (101.8-117.2)	102.0 (97 - 109)	88.5 (78.3-105.0)	1vs.2: <.001
				2vs.3: <.001
				1vs.3: <.001
PS median-IQR	77.4 (50.2 - 88.5)	49.3 (43.2 – 60.8)	77.5 (66.2-96.6)	1vs.2: <.001
				2vs.3: <.001
				1vs.3: .004

Both systolic and diastolic blood pressures were higher in the PE group as compared to the two control groups, p < 0.001. The PE group had significantly lower platelet counts than both control groups. Interestingly, the PE group had the highest PC levels among the three groups, whereas the non-pregnant group exhibited the highest PS levels, p < 0.001.

**Figure 1:**
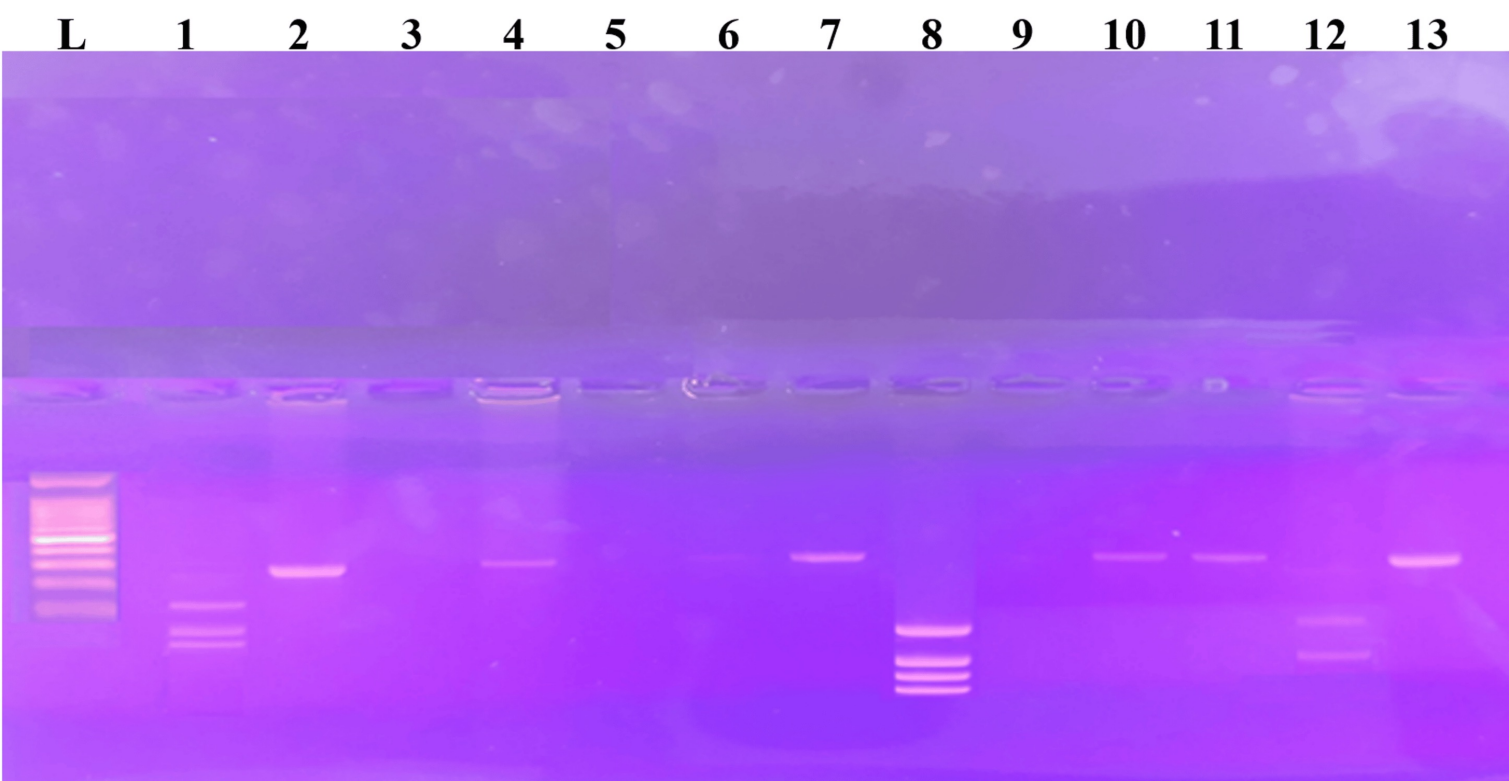
Polymerase chain reaction–restriction fragment length polymorphism (PCR-RFLP) analysis of Factor V Leiden (FVL) (R506Q) mutation by agarose gel electrophoresis Note: PCR-RFLP analysis for factor V Leiden (R506Q) polymorphism. Two fragments of 47,59 bp indicate mutant homozygous (A/A), three fragments of 136, 47, and 23bp indicate wild-type homozygous (G/G), and four fragments of 34, 47, 23, and 102 bp indicate heterozygous (A/G). Lane L shows the 100bp DNA ladder, Lane 1 wild type homozygous (G/G), Lane 2, 4, 6, 7, 11, 13 undigested sample of 206bp, Lane 3, 5 control with no DNA template, Lane 12 indicates homozygous mutant (A/A), and Lane 8 indicates heterozygous (A/G).

FVL (R506Q) genotypes were analyzed using polymerase chain reaction-restriction fragment length polymorphism (PCR-RFLP) and visualized with agarose gel electrophoresis, as shown in Figure 1. Although these differences were not statistically significant, the PE group had a higher proportion of homozygous positive (AA) individuals (8%) than the two control groups (3% each), as shown in Table 2.

**Table 2:** Distribution of FVL genotypes and alleles among study groups Note: Genotype comparisons were performed using chi-square tests. p < 0.05 indicates significance. FVL: Factor V Leiden

Genotype	Preeclampsia (N=100)	Healthy Pregnant (N=100)	Non-Pregnant (N=100)	Total (N=300)
Homozygous Positive FVL / AA	8%	3%	3%	14 (4.67%)
Heterozygous FVL / GA	0	2%	2%	4 (1.33%)
Negative FVL / GG	92%	95%	95%	282 (94%)
Allele A	16	8	8	32
Allele G	184	192	192	568

Table 3 outlines the distribution of thrombophilia markers across the three groups. The PE group had a significantly higher prevalence of low PC relative to the healthy pregnant group, p < 0.001, and displayed a higher frequency of low PS (31%) than the other groups. The combination of low PC and low PS was notably more frequent in the PE group than in controls, p < 0.001.

**Table 3:** Distribution of thrombophilia markers among PE cases, healthy pregnant controls, and non-pregnant controls Note: Chi-square tests are used for categorical comparisons. Significance set at p < 0.05. FVL: Factor V Leiden; PE: preeclampsia; PC: Protein C; PS: Protein S

	Cases (N=100)	Controls (N=200)		
	(1) Preeclampsia Group	(2) Healthy Pregnant Group	(3) Non-Pregnant Group	p-value
Positive FVL	8	5	5	1vs.2: .390
				2vs.3: >.999
				1vs.3: .390
Low PC	11	1	9	1vs.2: <.001
				2vs.3: .009
				1vs.3: .637
Low PS	31	65	1	1vs.2: .003
				2 vs.3 <.001
				1vs.3: .039
Combination of (Positive FVL + Low PS)	1	1	0	1vs.2: >.999
				2vs.3: .316
				1vs.3: .316
Combination of (Low PC + Low PS)	10	1	0	1vs.2: .005
				2vs.3: .316
				1vs.3: .001
Combination of (Positive FVL + Low PC + Low PS)	1	0	0	1vs.2: .316
				2vs.3: N/A
				1vs.3: .316
Combination of (Negative FVL, Normal PC, and Normal PS	62	31	85	1vs.2: <.001
				2vs.3: <.001
				1vs.3: <.001

The relationship between these thrombophilia markers and PE was further examined via logistic regression (Table 4). PS levels were correlated with PE (OR 1.07, p < 0.001), and the combination of low PC and PS displayed a synergistic effect (multivariate OR 77.67, 95% CI 8.97-672.5, p < 0.001).

**Table 4:** Thrombophilia markers and clinical variables in PE: univariate and multivariate analysis vs. healthy pregnant controls Note: Logistic regression analysis. p < 0.05 considered significant. FVL: Factor V Leiden; PE: preeclampsia; PC: Protein C; PS: Protein S; aOR: adjusted odds ratio; CI: confidence interval

Variable	Univariate OR (95% CI)	Univariate p-value	Multivariate aOR (95% CI)	Multivariate p-value
Consanguinity	1.09(0.61-1.96)	.765	—	—
Oral Contraceptive Pills	1.51(0.62-3.71)	.368	—	—
PC and PS				
PC Levels	1.01(0.99-1.02)	.419	—	—
PS Levels	1.07(1.04-1.09)	< .001>	1.09(1.06-1.11)	< .001>
Combination of Low PC + Low PS	11.00(1.38-87.61)	.024	77.67(8.97-672.5)	< .001>
FVL				
Positive FVL (Homo and Heterozygous)	1.65(0.52-5.24)	.394	—	—
Negative FVL	Reference			

## Discussion

This study examined the role of the natural blood clotting inhibitors PC and PS and the genetic polymorphism FVL in PE among Sudanese women.

Within this study, homozygous FVL was more common in the PE group (8%) than in the control group (5%), although the difference was not statistically significant (p=.390). Our results agree with a prior Sudanese study by Ahmed et al., which found a 9.6% prevalence of FVL among women with PE [7]. Globally, there was a great inconsistency in FVL rates: 0.76%, 8%, 15.4%, and 33% in Nigeria, Turkey, Egypt, and Ghana, respectively [2,17,18]. The prevalence of FVL in our control group was 5%, which is consistent with findings in regions such as Saudi Arabia (4.4%) [19], Egypt (2.5-10.2%), Tunisia (3.0-13.6%), Turkey (4.6-9.8%), the USA (3.2-6%), and Caucasian populations (3-8%) [20,21]. This variability underscores that the impact of FVL may differ across diverse genetic backgrounds.

Although FVL was more prevalent in the PE group, it was not significantly associated with the condition in both univariate and multivariate analyses. Several factors may explain this finding, including the relatively small sample size and possible interactions with other genetic or environmental variables. This suggests FVL may not be a major factor in PE within this multiethnic population, potentially due to intricate gene-environment interactions that diminish its impact [3]. Similarly, Changavala reported similar findings but found no significant link between FVL and PE [6]. However, other studies by Ahmed and Akhtar have indicated a potential association between FVL and PE in specific populations [7,22], emphasizing the population-dependent nature of this relationship.

PC levels were substantially higher in the PE group (11%) than in healthy pregnant controls (1%, p<.001). However, there was no significant difference between the PE group and non-pregnant controls (9%, p=.637). The median PC levels were also lower in the PE group than in both control groups (p<.001). PC was not identified as an independent predictor of PE by logistic regression (OR 1.01, 95% CI 0.99-1.02, p=.419). This is based on the results of Katz et al., who demonstrated that PC activity remains constant during pregnancy [23]. Research by Saleh et al. also reported reduced PC in PE but suggested that PC deficiency alone is not a strong predictor due to the complex interplay of coagulation factors during pregnancy [24].

Previous studies have suggested that low PS levels during pregnancy represent a normal physiological adaptation, particularly in the later stages of gestation [23,24]. The current findings showed that healthy pregnant women had lower PS levels than preeclamptic women. The reasons for this discrepancy may involve complex physiological or genetic factors unique to this study population. Additionally, the genetic diversity of the Sudanese population could contribute to variations in PS levels and their association with PE.

The PE group exhibited a substantially higher prevalence of the combination of low PC and low PS compared to both control groups (p<.001). This combination emerged as a strong predictor of PE in univariate and multivariate analyses. Although pregnancy typically enhances blood clotting to prevent hemorrhage during delivery, this adaptation can increase thrombotic risk in conditions such as PE, where clotting factors are dysregulated. These findings are supported by studies that underscore the importance of evaluating multiple clotting factors, as PC alone may not fully predict PE risk [24,25]. Jung et al. also linked decreased PS activity with hypertensive disorders of pregnancy [26], further corroborating the importance of combined PC/PS assessment.

The PE group exhibited a substantially higher prevalence of the combination of low PC and low PS compared to both control groups (p<.001). Consequently, this combination was a strong predictor of PE in both univariate and multivariate analyses. While pregnancy typically enhances blood clotting to prevent hemorrhage during delivery, this adaptation can increase the risk of blood clots in conditions such as PE, where clotting factors are disrupted. The increased risk of thrombosis observed in our study is corroborated by the stable role of PC as an anticoagulant and the decrease in PS levels [23]. Studies further emphasized the importance of measuring these proteins in states of increased blood clotting [24,25], while Jung et al. linked decreased PS activity with hypertensive disorders of pregnancy [26]. These studies support our findings that combined deficiencies in PC/PS indicate a higher risk of PE.

Consanguinity (marriage between relatives) and contraceptive use were not significant predictors of PE in this study. Although consanguinity was prevalent in both groups, particularly among those with PE, the difference was not statistically significant, paralleling other work [27]. However, different studies suggest that consanguinity can increase genetic risks for PE by passing on recessive alleles [11,14,28]. It is plausible that larger or multicenter studies might detect subtler genetic predispositions associated with consanguinity. Similarly, hormonal contraceptive use was not a significant factor for PE, which is consistent with prior research [29,30].

Despite the informative nature of these findings, certain study characteristics may influence their broader applicability. For instance, the number of participants included was relatively modest, which could limit direct extrapolation to other populations. Additionally, a more in-depth assessment of factors such as nutrition, socioeconomic status, and underlying comorbidities might yield further insights into how coagulation profiles vary. Overall, these results underscore the multifactorial nature of PE, likely involving a complex interplay of genetic and environmental determinants. While FVL alone may not substantially contribute to PE risk in this population, combined deficiencies in PC and PS levels appear to play a more pivotal role.

## Conclusions

Combined deficiencies in PC and PS levels are strongly associated with a higher risk of PE among Sudanese women, indicating that multiple natural anticoagulant pathways may be involved in PE pathogenesis. In contrast, FVL mutation was not significantly associated with PE in this Sudanese population. These data underscore the importance of a multifactorial evaluation of thrombophilia when assessing PE risk in diverse populations.
